# The determinants and trajectories of transport disadvantage among American older adults

**DOI:** 10.1093/geroni/igaf122.375

**Published:** 2025-12-31

**Authors:** Yong Yang

**Affiliations:** The University of Memphis, Memphis, Tennessee, United States

## Abstract

**Background:**

Maintaining daily transportation becomes increasingly challenging with age due to various barriers. Transport disadvantage—defined as inadequate access to transportation via car, public transit, or other modes—remains understudied in older adulthood. Little is known about how it evolves over time, whether it accelerates or stabilizes at different aging stages, and how trajectories vary among older adults.

**Methods:**

This study examines changes in transport disadvantage from 2011 to 2024 using data from the National Health and Aging Trends Study (NHATS), a nationally representative panel study of U.S. Medicare enrollees aged 65 and older. We apply linear mixed-effects models and group-based trajectory models to assess transport disadvantage over time, identify distinct trajectories, and examine the influence of personal characteristics (e.g., demographics, health status) and neighborhood conditions (e.g., deprivation, safety, walkability, public transit access).

**Results:**

We identify three trajectories: (1) a majority with no transport restrictions, (2) a group with low but stable or increasing disadvantage, and (3) a group with moderate to high disadvantage that declines over time. Transport disadvantage generally increases with age, with a sharp decline following driving cessation. Older adults in high-deprivation, low-safety, or poor-transit neighborhoods are more likely to experience worsening transport disadvantage, with effects amplified among those with disabilities, lower socioeconomic status, or declining health.

**Conclusion:**

Addressing disparities in transport access requires targeted interventions that consider both individual vulnerabilities and neighborhood factors to support mobility and well-being in aging populations.

